# Supplementary material for: COZOID: contact zone identifier for visual analysis of protein-protein interactions
Source: BMC Bioinformatics. 2018 Apr 6;19:125. doi: 10.1186/s12859-018-2113-6 (PMC5889581; doi:10.1186/s12859-018-2113-6)
Supplement: Supplementary file 2 — Software build. Executable binary file of the software tool. (ZIP 118,784 kb) [file 12859_2018_2113_MOESM2_ESM.zip › cozoid/java/docs/beansbinding-1.2.1-doc.zip!/org/jdesktop/el/impl/lang/ELSupport.html]

ELSupport (Beans Binding)


---


|  |  |  |
| --- | --- | --- |
| **Overview** \| **Package** \| **Class** \| **Use** \| **Tree** \| **Deprecated** \| **Index** \| **Help** | |  |
| **PREV CLASS**   **NEXT CLASS** | **FRAMES**    **NO FRAMES**     **All Classes** |  |
| SUMMARY: NESTED \| FIELD \| CONSTR \| METHOD | DETAIL: FIELD \| CONSTR \| METHOD |  |


---


## org.jdesktop.el.impl.lang Class ELSupport

```
java.lang.Object
  org.jdesktop.el.impl.lang.ELSupport
```

**Direct Known Subclasses:**: SimpleNode

---

``` public class ELSupport extends java.lang.Object ```

A helper class that implements the EL Specification

---

**Constructor Summary**

`ELSupport()`


| **Method Summary** | |
| --- | --- |
| `static java.lang.Boolean` | `coerceToBoolean(java.lang.Object obj)` |
| `static java.lang.Character` | `coerceToCharacter(java.lang.Object obj)` |
| `static java.lang.Enum` | `coerceToEnum(java.lang.Object obj, java.lang.Class type)` |
| `protected static java.lang.Number` | `coerceToNumber(java.lang.Number number, java.lang.Class type)` |
| `static java.lang.Number` | `coerceToNumber(java.lang.Object obj)` |
| `static java.lang.Number` | `coerceToNumber(java.lang.Object obj, java.lang.Class type)` |
| `protected static java.lang.Number` | `coerceToNumber(java.lang.String val, java.lang.Class type)` |
| `static java.lang.String` | `coerceToString(java.lang.Object obj)` |
| `static java.lang.Object` | `coerceToType(java.lang.Object obj, java.lang.Class type)` |
| `static int` | `compare(java.lang.Object obj0, java.lang.Object obj1)` |
| `static boolean` | `containsNulls(java.lang.Object[] obj)` |
| `static boolean` | `equals(java.lang.Object obj0, java.lang.Object obj1)` |
| `static boolean` | `isBigDecimalOp(java.lang.Object obj0, java.lang.Object obj1)` |
| `static boolean` | `isBigIntegerOp(java.lang.Object obj0, java.lang.Object obj1)` |
| `static boolean` | `isDoubleOp(java.lang.Object obj0, java.lang.Object obj1)` |
| `static boolean` | `isDoubleStringOp(java.lang.Object obj0, java.lang.Object obj1)` |
| `static boolean` | `isLongOp(java.lang.Object obj0, java.lang.Object obj1)` |
| `static boolean` | `isStringFloat(java.lang.String str)` |
| `static void` | `throwUnhandled(java.lang.Object base, java.lang.Object property)` |
| `static java.lang.Number` | `toFloat(java.lang.String value)` |
| `static java.lang.Number` | `toNumber(java.lang.String value)` |

**Methods inherited from class java.lang.Object**

`clone, equals, finalize, getClass, hashCode, notify, notifyAll, toString, wait, wait, wait`

**Constructor Detail**

### ELSupport

```
public ELSupport()
```


**Method Detail**

### throwUnhandled

```
public static final void throwUnhandled(java.lang.Object base,
                                        java.lang.Object property)
                                 throws ELException
```

:   **Throws:**: `ELException`

---


### compare

```
public static final int compare(java.lang.Object obj0,
                                java.lang.Object obj1)
                         throws ELException
```

:   **Parameters:**: `obj0` -: `obj1` - **Returns:**: **Throws:**: `EvaluationException`: `ELException`

---


### equals

```
public static final boolean equals(java.lang.Object obj0,
                                   java.lang.Object obj1)
                            throws ELException
```

:   **Parameters:**: `obj0` -: `obj1` - **Returns:**: **Throws:**: `EvaluationException`: `ELException`

---


### coerceToBoolean

```
public static final java.lang.Boolean coerceToBoolean(java.lang.Object obj)
                                               throws java.lang.IllegalArgumentException
```

:   **Parameters:**: `obj` - **Returns:**: **Throws:**: `java.lang.IllegalArgumentException`

---


### coerceToEnum

```
public static final java.lang.Enum coerceToEnum(java.lang.Object obj,
                                                java.lang.Class type)
                                         throws java.lang.IllegalArgumentException
```

:   **Throws:**: `java.lang.IllegalArgumentException`

---


### coerceToCharacter

```
public static final java.lang.Character coerceToCharacter(java.lang.Object obj)
                                                   throws java.lang.IllegalArgumentException
```

:   **Throws:**: `java.lang.IllegalArgumentException`

---


### coerceToNumber

```
public static final java.lang.Number coerceToNumber(java.lang.Object obj)
```

---


### coerceToNumber

```
protected static final java.lang.Number coerceToNumber(java.lang.Number number,
                                                       java.lang.Class type)
                                                throws java.lang.IllegalArgumentException
```

:   **Throws:**: `java.lang.IllegalArgumentException`

---


### coerceToNumber

```
public static final java.lang.Number coerceToNumber(java.lang.Object obj,
                                                    java.lang.Class type)
                                             throws java.lang.IllegalArgumentException
```

:   **Throws:**: `java.lang.IllegalArgumentException`

---


### coerceToNumber

```
protected static final java.lang.Number coerceToNumber(java.lang.String val,
                                                       java.lang.Class type)
                                                throws java.lang.IllegalArgumentException
```

:   **Throws:**: `java.lang.IllegalArgumentException`

---


### coerceToString

```
public static final java.lang.String coerceToString(java.lang.Object obj)
```

:   **Parameters:**: `obj` - **Returns:**

---


### coerceToType

```
public static final java.lang.Object coerceToType(java.lang.Object obj,
                                                  java.lang.Class type)
                                           throws java.lang.IllegalArgumentException
```

:   **Throws:**: `java.lang.IllegalArgumentException`

---


### containsNulls

```
public static final boolean containsNulls(java.lang.Object[] obj)
```

:   **Parameters:**: `obj` - **Returns:**

---


### isBigDecimalOp

```
public static final boolean isBigDecimalOp(java.lang.Object obj0,
                                           java.lang.Object obj1)
```

---


### isBigIntegerOp

```
public static final boolean isBigIntegerOp(java.lang.Object obj0,
                                           java.lang.Object obj1)
```

---


### isDoubleOp

```
public static final boolean isDoubleOp(java.lang.Object obj0,
                                       java.lang.Object obj1)
```

---


### isDoubleStringOp

```
public static final boolean isDoubleStringOp(java.lang.Object obj0,
                                             java.lang.Object obj1)
```

---


### isLongOp

```
public static final boolean isLongOp(java.lang.Object obj0,
                                     java.lang.Object obj1)
```

---


### isStringFloat

```
public static final boolean isStringFloat(java.lang.String str)
```

---


### toFloat

```
public static final java.lang.Number toFloat(java.lang.String value)
```

---


### toNumber

```
public static final java.lang.Number toNumber(java.lang.String value)
```


---


|  |  |  |
| --- | --- | --- |
| **Overview** \| **Package** \| **Class** \| **Use** \| **Tree** \| **Deprecated** \| **Index** \| **Help** | |  |
| **PREV CLASS**   **NEXT CLASS** | **FRAMES**    **NO FRAMES**     **All Classes** |  |
| SUMMARY: NESTED \| FIELD \| CONSTR \| METHOD | DETAIL: FIELD \| CONSTR \| METHOD |  |


---
